# Metformin Suppresses Self-Renewal Ability and Tumorigenicity of Osteosarcoma Stem Cells via Reactive Oxygen Species-Mediated Apoptosis and Autophagy

**DOI:** 10.1155/2019/9290728

**Published:** 2019-11-18

**Authors:** Bin Zhao, Jie Luo, Ye Wang, Liangfu Zhou, Jingmin Che, Fang Wang, Songlin Peng, Ge Zhang, Peng Shang

**Affiliations:** ^1^Research & Development Institute of Northwestern Polytechnical University in Shenzhen, Shenzhen 518057, China; ^2^School of Life Science, Northwestern Polytechnical University, Xi'an, Shaanxi 710072, China; ^3^Department of Spine Surgery, Shenzhen People' Hospital, Shenzhen 518000, China; ^4^Institute of Integrated Bioinfomedicine and Translational Science, School of Chinese Medicine, Hong Kong Baptist University, Hong Kong 999077, China; ^5^Key Laboratory for Space Bioscience and Biotechnology, Institute of Special Environmental Biophysics, Northwestern Polytechnical University, Xi'an, Shaanxi 710072, China

## Abstract

Osteosarcoma is the most frequently diagnosed primary malignant bone sarcoma in children and adolescents. Recent studies have shown that cancer stem cells (CSCs), a cluster of tumor cells with the ability to self-renew, play an essential role in tumor recurrence and metastasis. Thus, it is necessary to develop therapeutic strategies specifically targeting CSCs. Metformin, the first-line drug for type 2 diabetes, exhibits antineoplastic activities in various kinds of tumors. New evidence has suggested that metformin may target CSCs and prevent their recurrence. However, the underlying specific mechanisms remain unclear. In this study, we found that metformin significantly suppressed the self-renewal ability of osteosarcoma stem cells (OSCs) and induced G0/G1 phase arrest by blocking the activity of cyclin-dependent kinases. Furthermore, metformin induced apoptosis through a mitochondria-dependent pathway, leading to the collapse of the mitochondrial transmembrane potential and the production of reactive oxygen species (ROS). Importantly, metformin acted directly on the mitochondria, which resulted in decreased ATP synthesis. This change allowed access to the downstream AMPK kinase, and the activation of AMPK led to the reversal of the mTOR pathway, triggering autophagy. Particularly, metformin-mediated autophagy disturbed the homeostasis of stemness and pluripotency in the OSCs. Additionally, our mouse xenograft model confirmed the potential therapeutic use of metformin in targeting OSCs. In conclusion, our findings suggest that metformin suppresses the self-renewal ability and tumorigenicity of OSCs via ROS-mediated apoptosis and autophagy.

## 1. Introduction

Osteosarcoma, the most frequently diagnosed primary malignant tumor in children and adolescents, is characterized by a high risk of developing lung metastasis and poor prognosis [1]. Despite steady progress in the research and development of cancer therapeutics, the 5-year survival rates of osteosarcoma remain the same as they were in the 1970s [2]. Cancer stem cells (CSCs), a subgroup of cancer cells with the ability to self-renew, play an essential role in tumor recurrence and metastasis [3]. Study has showed that the key challenge of cancer was the late occurrence of distant metastases, in which CSCs played a dominant role [4]. Therefore, targeting CSCs may be a promising strategy for the future of cancer treatment.

Metformin, the most widely used drug in the treatment of type 2 diabetes, exerted antineoplastic activities on a vast majority of cancers [5–7]. Moreover, numerous studies have indicated that metformin is a promising therapy against CSCs [8–10]. Autophagy, an essential homeostatic process for protein degradation, was recently identified as a crucial regulator in tumorigenesis [11]. Further research suggested that autophagy may regulate drug sensitivity and the pluripotency of CSCs [12]. However, the roles that autophagy plays in CSCs are largely unknown. Understanding these roles, as well as the molecular mechanisms underlying the maintenance of CSCs, may be critical for oncotherapy.

In this study, osteosarcoma stem cells (OSCs) from K7M2 and MG63 osteosarcoma cell lines were isolated by methods of both side population (SP) analysis and serum-free suspension culture. Both K7M2 and MG63 OSCs expressed high levels of stem cell markers including Sox2, Oct4, Nanog, CD44, CD133, and ALDH1. Also, both K7M2 and MG63 OSCs had the ability to differentiate into osteogenic and chondrogenic lineages. Notably, metformin treatment induced cell cycle arrest and decreased the viability of both the K7M2 and MG63 OSCs. In addition, metformin triggered apoptosis in both K7M2 and MG63 OSCs via a caspase-dependent mitochondrial apoptotic pathway, which was associated with alterations in the morphology of the mitochondrial structure and the balance of mitochondrial Bcl-2 and Bax. Most importantly, we found that autophagic flux was higher in these OSCs than it was in their parental cells. Treatment with metformin abrogated the pluripotency in the K7M2 and MG63 OSCs associated with the upregulation of autophagy through the AMPK/mTOR pathway. When the OSCs were treated with 3-methyladenine (3MA), an inhibitor of autophagy, there was a decrease in their stemness properties. Likewise, treatment with the autophagy inducer rapamycin also impaired the pluripotency of both OSCs, abolishing their stemness. Additionally, the therapeutic efficacy of metformin was further confirmed in mice bearing K7M2 osteosarcoma xenografts. This study suggests that metformin has promising anticancer activity in OSCs through regulation of autophagy.

## 2. Material and Methods

### 2.1. Drugs, Reagents, and Antibodies

Metformin (D150959), Hoechst 33342 (14533), verapamil (V4629), 3MA (189490), and rapamycin (553210) were purchased from Sigma-Aldrich (Saint Louis, USA). The cell mitochondria isolation kit (C3601), Reactive Oxygen Species Assay Kit (S0033), FITC-Annexin V/PI apoptosis detection kit (C1062L), ATP assay kit (S0026), and anti-p21 (AP021), anti-Cyclin D1 (AC853), anti-Cyclin D3 (AC856), anti-ATG5 (AF2269), anti-ATG7 (AA820), and anti-Nanog (AF1912) antibodies were purchased from Beyotime (Haimen, China). The anti-Bcl-2 (ab182858), anti-Bax (ab182733), anti-active Caspase9 (ab2324), anti-active Caspase3 (ab2302), anti-cytochrome c (ab133504), anti-LC3 (ab48394), anti-Oct4 (ab181557), anti-Sox2 (ab97959), anti-Ki67 (ab15580), anti-AMPK (ab80039), anti-phospho AMPK (ab133448), anti-mTOR (ab134903), anti-phospho mTOR (ab109268), CD105 (ab135528), and Stro-1 (ab106531) antibodies were obtained from Abcam (USA). The anti-*β*-actin (4967S and E4D9Z) was purchased from Cell Signaling Technology (Danvers, USA). Mito Tracker® Red CMXRos (M7512) and Lyso Tracker Red DND-99 (L7528) were obtained from Thermo Fisher Scientific (Waltham, USA). Alexa Fluor® 488 mouse/human anti-Sox2 antibody (656109), PE anti-mouse/human Oct4 antibody (653703), APC anti-mouse/human CD44 antibody (103011), APC anti-mouse CD133 antibody (141207), and PE/Cy7 anti-human CD133 antibody (372809) were obtained from Biolegend (San Diego, USA). Acridine orange/ethidium bromide (AO/EB) was from Solarbio (Beijing, China). DAPI (BD5010) was obtained from Bioworld Technology, Inc. DMEM (12800082), DMEM/F12 (12400024), Fetal Bovine Serum (FBS, 10099-141), B27 Supplement (17504044), epidermal growth factor (EGF, PHG0311), and basic fibroblast growth factor (bFGF, 13256029) were purchased from Gibco (USA).

### 2.2. Animals

Four-week-old male Balb/c mice were obtained from the Fourth Military Medical University. All experimental procedures were conducted under the protocol reviewed and approved by the Ethics Committee of the Northwestern Polytechnical University.

### 2.3. Cell Viability Assay

The viability of the K7M2 and MG63 OSCs was evaluated by CCK-8 assay. Briefly, cultured K7M2 and MG63 OSCs were seeded into 96-well microtiter plates at a density of 1 × 10^4^ cells/well and treated with different concentrations of metformin (0, 6.4, 12.8, 25.6, and 51.2 mM) for 24-72 h. The cells were then treated with CCK-8 solution, and the absorbances were measured by multiscan spectrum (Bio-Rad, USA) at 570 nm. The cell death of the K7M2 and MG63 OSCs was also determined by staining with AO/PI for 10 min. Images were taken using a ZEISS inversion fluorescence microscope (Zeiss, Germany).

### 2.4. Cell Cycle Analysis

The cell cycle distribution of the K7M2 and MG63 OSCs was assessed by flow cytometry. Briefly, after 48 h of metformin (0, 6.4, 12.8, 25.6, and 51.2 mM) treatment, the K7M2 and MG63 OSCs were harvested and made into single cell suspensions by mechanical blow method. The cells were then fixed, centrifuged, resuspended, stained with 50 mg/mL of PI and 0.5 mg/mL of RNase A for 30 min in the dark, and then analyzed by flow cytometry (BD FACS Calibur, USA).

### 2.5. Apoptosis Assay

The K7M2 and MG63 OSCs were seeded into 90 mm culture plates at a density of 1 × 10^6^ cells/well and treated with metformin (0, 6.4, 12.8, 25.6, and 51.2 mM) for 48 h. The cells were harvested and labeled with FITC-Annexin V/PI at room temperature for 20 min according to the instructions. The samples were then analyzed using flow cytometry (BD FACS Calibur, USA).

### 2.6. Side Population (SP) Assay

Flow cytometry was used to identify the SP fractions as described by Steiniger et al. [13]. In brief, cultured cells were trypsinized and resuspended in prewarmed DMEM prior to incubation with 5 *μ*g/mL Hoechst 33342 either alone or in the presence of 50 *μ*g/mL of the ABC transporter inhibitor verapamil for 90 min at 37°C. The cells were then passed through a 100 *μ*m mesh filter (BD, USA) and sorted by a FACS Aria III flow cytometer (BD, USA) equipped with Hoechst Blue with a 375 broad pass filter and Hoechst Red with a 675 broad pass filter laser. The sorted SP cells were collected and fluorescent-labeled with Sox2, Oct4, CD44, CD133, CD105, and Stro-1 to detect their stemness by a flow cytometer.

### 2.7. Aldehyde Dehydrogenase (ALDH1) Assay

K7M2 and MG63 OSCs were treated with 6.4 mM metformin, 0.5 mM 3MA, 6.4 mM metformin, and 0.5 mM 3MA together, or 5 *μ*M rapamycin for 48 h. The ALDH1-positive subpopulation was analyzed using an ALDEFLUOR assay kit (Stem Cell Technologies, Canada). Approximately 1 × 10^6^ K7M2 and MG63 OSCs were collected and incubated in the ALDEFLUOR assay buffer for 40 min at 37°C. Negative control samples were treated with 50 *μ*M of diethylaminobenzaldehyde (DEAB), an inhibitor of ALDH1. Following that, the cells were harvested and resuspended in ALDEFLUOR buffer and subjected to the FACS Aria III flow cytometer.

### 2.8. Mitochondrial Membrane Depolarization

Mitochondrial membrane potential was monitored by Mito flow fluorescent dye (Cell Technology Inc., USA). Briefly, approximately 5 × 10^5^ K7M2 and MG63 OSCs were incubated with the indicated concentrations of metformin (0, 6.4, 12.8, 25.6, and 51.2 mM) for 48 h. The cells were then stained with fluorescent dye for 30 min and detected by a BD FACS Calibur flow cytometer.

### 2.9. Reactive Oxygen Species (ROS) Detection

ROS were detected by measuring the oxidation of dichloro-dihydro-fluorescein diacetate (DCFH-DA) using Reactive Oxygen Species Assay Kit. Cells at 50%-60% confluency were incubated with metformin (0, 6.4, 12.8, 25.6, and 51.2 mM) for 48 h and then treated with 10 *μ*M of DCFH-DA for 30 min at 37°C in the dark. The cells were then washed and harvested prior to analysis using the FACS Aria III flow cytometer.

### 2.10. Measurement of Intracellular ATP

Intracellular ATP content was determined using an ATP assay kit according to the manufacturer's instructions. In brief, K7M2 and MG63 OSCs were planted on 96-well plates and treated with metformin (0, 6.4, 12.8, 25.6, and 51.2 mM) for 48 h. The cells were lysed with an ATP extraction buffer and centrifuged to collect the supernatant. The supernatant was then mixed with the dilution buffer containing luciferase and measured by multiscan spectrum (Bio-Rad, USA).

### 2.11. Real-Time PCR

Total RNA was extracted from the metformin-treated OSCs by TRIzol reagent (Invitrogen, USA). The isolated RNA was reverse transcribed into cDNA using the Prime Script RT Reagent kit (Takara Biotechnology, China). Primers were obtained from Sangon Biotech (Shanghai, China). Quantitative PCR was performed using an IQ5 real-time PCR system (Bio-Rad, USA) in a 20 *μ*L reaction volume. The sequences of primers are listed as follows: human *P21*: 5′-AGCAGCGGAACAAGGAGT-3′ (sense) and 5′-CGTTAGTGCCAGGAAAGACA-3′ (antisense); mouse *P21*: 5′-GACAAGAGGCCCAGTACTTC-3′ (sense) and 5′-TAGAAATCTGTCAGGCTGGT-3′ (antisense); human *Cyclin D1*: 5′-TCTCCAAAATGCCAGAGGCG-3′ (sense) and 5′-AGGAAGTTGTTGGGGCTCCT-3′ (antisense); mouse *Cyclin D1*: 5′-CGGATGAGAACAAGCAGACC-3′ (sense) and 5′-GCAGGAGAGGAAGTTGTTGG-3′ (antisense); human *Cyclin D3*: 5′-AGGGATCACTGGCACTGAAG-3′ (sense) and 5′-ACAGGTGTATGGCTGTGACAT-3′ (antisense); mouse *Cyclin D3*: 5′-CTATGAACTACCTGGATCGCTACCT-3′ (sense) and 5′-CAGACGGTACCTAGAAGCTGCAA-3′ (antisense); human *SOX2*: 5′-AACCCCAAGATGCACAACTC-3′ (sense) and 5′-CGGGGCCGGTATTTATAATC-3′ (antisense); mouse *SOX2*: 5′-GCGGAGTGGAAACTTTTGTCC-3′ (sense) and 5′-GGGAAGCGTGTACTTATCCTTCT-3′ (antisense); human *OCT4*: 5′-GCTCGAGAAGGATGTGGTCC-3′ (sense) and 5′-CGTTGTGCATAGTCGCTGCT-3′ (antisense); mouse *OCT4*: 5′-CGGAAGAGAAAGCGAACTAGC-3′ (sense) and 5′-ATTGGCGATGTGAGTGATCTG-3′ (antisense); human *NANOG*: 5′-CAAAGGCAAACAACCCACTT-3′ (sense) and 5′-TCTGCTGGAGGCTGAGGTAT-3′ (antisense); mouse *NANOG*: 5′-TGACCTCAACTACATGGTCTACA-3′ (sense) and 5′-CTTCCCATTCTCGGCCTTG-3′ (antisense); human *ATG5*: 5′-CACA AGCAACTCTGGATGGGATT-3′ (sense) and 5′-CCATCTTCAG GATCAATAGCAGAAG-3′ (antisense); mouse *ATG5*: 5′-GTGCTTCGAGATGTGTGGTTTGGA-3′ (sense) and 5′-CGTCAAATAGCTGACTCTTGGCAA-3′ (antisense); human *ATG7*: 5′-GGTCAAAGGACGAAGATAACA-3′ (sense) and 5′-GGTCACGGAAGCAAACAACT-3′ (antisense); mouse *ATG7*: 5′-GCTAATGGACACCAGGGAGA-3′ (sense) and 5′-AAAAAGTGAGGAGCCCAGGT-3′ (antisense); human *GAPDH*: 5′-TTGATGGCAACAATCTCCAC-3′ (sense) and 5′-CGTCCCGTAGACAAAATGGT-3′ (antisense); and mouse *GAPDH*: 5′-CAACAGCAACTCCCACTCTTC-3′ (sense) and 5′-GGTCCAGGGTTTCTTACTCCTT-3′ (antisense).

### 2.12. Western Blot

Mitochondria were extracted by the mitochondria isolation kit according to the manufacturer's instructions. The isolated mitochondria or cells were lysed with RIPA buffer containing protease inhibitor or phosphatase inhibitor (Beyotime, China) and determined by bicinchoninic acid assay (Beyotime, China). Thirty micrograms of protein was loaded onto SDS-PAGE, separated by electrophoresis, and transferred to polyvinylidene difluoride membrane (PVDF). The membranes were blocked with 1% bovine serum albumin (BSA), incubated with primary antibodies, washed with TBST buffer, and then incubated with HRP-conjugated secondary antibodies (Boster, China). Immunoblot images were taken and quantified using ImageJ software (National Institutes of Health, USA). The intensities of the bands were determined and normalized to *β*-actin.

### 2.13. Tumor Sphere Assay

K7M2 and MG63 OSCs were treated with 6.4 mM metformin, 0.5 mM 3MA, 6.4 mM metformin, and 0.5 mM 3MA together, or 5 *μ*M rapamycin for 48 h. Tumor spheres were induced by 6-well ultralow adherent culture plates (Corning, USA). K7M2 and MG63 cells were seeded at a density of 5 × 10^3^ cells/well in a serum-free DMEM/F12 medium supplemented with 1× B27, 10 ng/mL EGF, and 10 ng/mL bFGF. Tumor sphere formation was quantified 7 days after initial seeding by staining with 0.1% crystal violet (Sigma-Aldrich, USA) and imaged with an inverted microscope (Olympus, Japan).

### 2.14. Immunohistochemistry (IHC) and Immunofluorescence (IF)

For IHC, tumor tissues were harvested, fixed, dehydrated, embedded in paraffin, and sliced into 4 *μ*m sections. The slices were then stained with hematoxylin-eosin (H&E) and IHC against LC3, ATG5, Ki67, Sox2, and Oct4. For IF, the cells were fixed with 4% paraformaldehyde, blocked with 1% BSA, and then incubated in primary antibodies overnight at 4°C. The cells were then washed with phosphate-buffered saline (PBS) and incubated with fluorescence-labeled secondary antibodies at room temperature in the dark. Images were taken using an FSX100 microscope (Olympus, Japan) or an FV10i Confocal Laser Scanning Microscope (Olympus, Japan).

### 2.15. Differentiation Potential of OSCs

The K7M2 and MG63 OSCs treated with/without metformin were cultured in a commercial osteogenic and chondrogenic differentiation medium (Cyagen, China) in 6-well cell culture plates for three weeks. The cells were then fixed with 4% paraformaldehyde and stained with Alizarin Red (Cyagen, China) for detection of osteogenic differentiation and Alcian Blue (Cyagen, China) for chondrogenic differentiation. Images were taken using an FSX100 microscope (Olympus, Japan).

### 2.16. Transmission Electron Microscope (TEM)

For TEM detection, cells with different treatments were fixed with 2% phosphotungstic acid, dropped onto formvar/carbon-coated copper mesh grids, and then left to dry at room temperature. Images were taken with a Hitachi HT7700 transmission electron microscope (Hitachi, Japan).

### 2.17. Scanning Electron Microscope (SEM)

OSCs were fixed with 2.5% glutaraldehyde, dehydrated in ethanol, dried at the critical point, and then placed on copper grids. The specimens were then imaged using a Hitachi SU8010 scanning electron microscope (Hitachi, Japan).

### 2.18. Orthotopic Intratibial Mouse Model of Osteosarcoma

After pretreatment of 6.4 mM metformin for 48 h, approximately 1 × 10^5^, 1 × 10^4^, or 1 × 10^3^ of the K7M2 OSCs were suspended in 30 *μ*L PBS and implanted into the tibia of male Balb/c mice by intratibial injection. Mice were anesthetized, the left leg was held with the knee, and the needle was inserted into the tibial tuberosity using a drilling motion. After 2 weeks, the mice were euthanized, and the tumor volumes and weights were measured (tumor volume = (length × width × height)/2). To further assess the effect of metformin-mediated autophagy on tumorigenesis, approximately 1 × 10^5^ of K7M2 OSCs suspended in 30 *μ*L PBS were injected into the tibia of male Balb/c mice. When tumor volumes reached about 100 mm^3^, the mice were then randomly divided into five groups, receiving intraperitoneal injection of (1) PBS, (2) 250 mg/kg/day metformin diluted with PBS [14], (3) 15 mg/kg/day 3MA diluted with PBS [15], (4) 250 mg/kg metformin + 15 mg/kg/day 3MA, and (5) 1 mg/kg/day rapamycin diluted with PBS [16]. The mice were killed after 21 days, and the tumors were removed, weighed, and subjected to IHC staining for LC3, ATG5, Ki67, Sox2, and Oct4.

### 2.19. Statistical Analysis

Data analysis was performed using GraphPad Prism (GraphPad Software Inc., USA). Statistical analysis was performed by independent samples *t*-test for comparison between two groups or one-way ANOVA among the groups. *P* < 0.05 was considered statistically significant.

## 3. Results

### 3.1. Autophagic Flux Was Enhanced in OSCs

**Figure 1:**
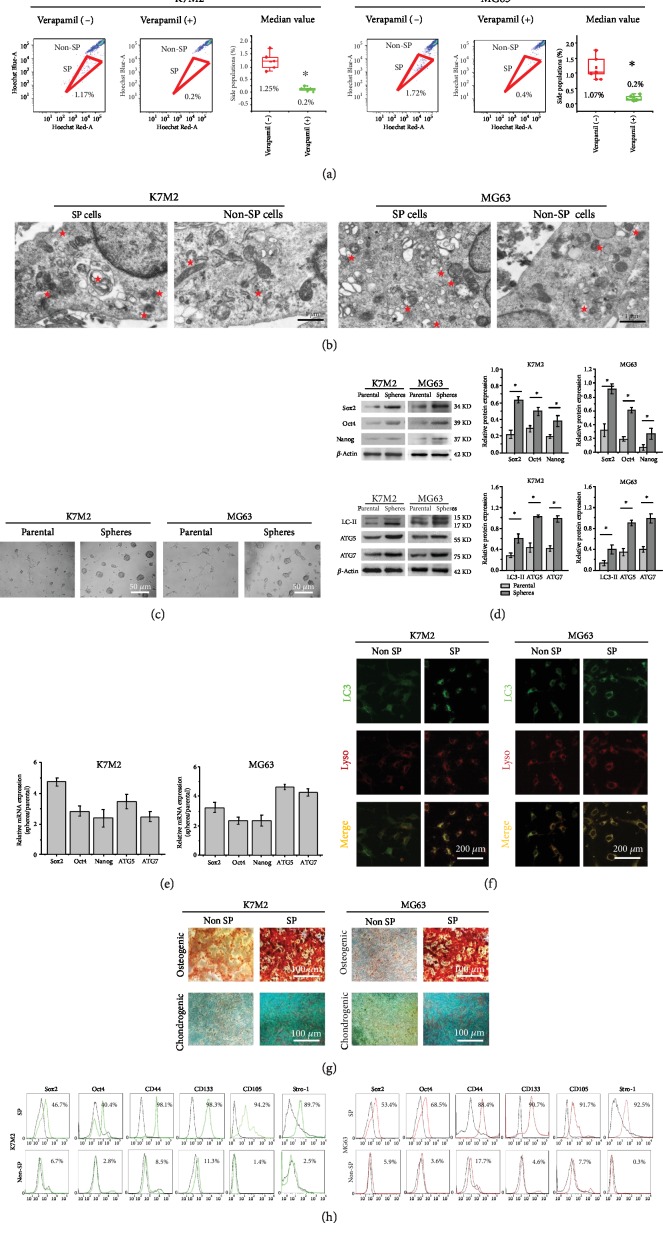
Characteristics of OSCs. (a) The representative images of SP cells from K7M2 and MG63 osteosarcoma cell lines. The median value of K7M2 and MG63 SP cells was 1.25% and 1.07%, respectively. *n* = 5. (b) The representative TEM images of autophagosomes in K7M2 and MG63 SP cells. The pentagrams stand for autophagosomes. Scale bars = 1 *μ*m. (c) Tumor spheres of K7M2 and MG63 osteosarcoma cells after culturing in the serum-free medium DMEM/F12-bFGF-EGF-B27 for 7 days. The parental K7M2 and MG63 cells cultured in DMEM/F12 supplemented with 1% FBS served as a control. Scale bars = 50 *μ*m. *n* = 5. (d) Western blot analysis of the pluripotent transcription factors Sox2, Oct4, and Nanog and the autophagy markers LC3, ATG5, and ATG7 in K7M2 and MG63 OSCs. Data are shown as mean ± SD, *n* = 3. (e) The mRNA expression levels of the pluripotency-associated genes *SOX2*, *OCT4*, and *NANOG* and the autophagy-related genes *ATG5* and *ATG7*. *n* = 3. (f) Immunofluorescence analysis of autophagy in K7M2 and MG63 SP cells. The colocalization (orange) staining of LC3 (green) with lysosome (red) indicates autophagy. Scale bars = 200 *μ*m. *n* = 3. (g) Osteogenic and chondrogenic differentiation of K7M2 and MG63 SP cells. Cells differentiated into osteoblasts and chondroblasts were detected by staining with Alizarin Red and Alcian Blue. Scale bars = 100 *μ*m. *n* = 3. (h) Flow cytometry-based assay for the pluripotent transcription factors Sox2 and Oct4 and the CSC surface markers CD44, CD105, CD133, and Stro-1 in K7M2 and MG63 SP cells. *n* = 3. ^∗^*P* < 0.05 was considered statistically significant.

We isolated OSCs from K7M2 and MG63 osteosarcoma cell lines of which side population (SP) phenotype revealed as a characteristic tail separated from the complete population in Figure 1(a). The median percentage of K7M2 and MG63 SP cells was 1.25% and 1.07%, respectively, and the SP cells decreased to 0.2% and 0.2% upon treatment with verapamil, the inhibitor of the ABC transporter. To determine whether the basal level of autophagic flux was different between general osteosarcoma cells and their OSCs, we first observed the autophagosomes by TEM. As shown in Figure 1(b), the numbers of autophagosomes were significantly increased in SP cells than in non-SP cells, indicating that K7M2 and MG63 OSCs have a higher basal autophagic flux. Moreover, OSCs from K7M2 and MG63 osteosarcoma cells were successfully isolated via serum-free suspension culture for 7 days, and the tumor spheres were formed as showed in Figure 1(c). To further characterize the spheres and their parental cells, the stemness and autophagic properties were studied. As shown in Figure 1(d), the sphere cells from both K7M2 and MG63 had higher protein expression levels of the pluripotent transcription factors including Sox2, Oct4, and Nanog, as well as the high levels of autophagy-associated proteins LC3-II, ATG5, and ATG7. Real-time PCR (Figure 1(e)) also revealed that the sphere cells of both K7M2 and MG63 had higher mRNA levels of the pluripotent genes *SOX2*, *OCT4*, and *NANOG* and the autophagy-related genes *ATG5* and *ATG7*. Immunofluorescent staining assay (Figure 1(f)) confirmed that both K7M2 and MG63 SP cells have stronger fluorescent punctate structures of LC3-II than their non-SP cells, indicating higher levels of autophagy in OSCs. To determine the differentiation ability of the SP cells, they were cultured in an adhesive culture system in an osteogenic and chondrogenic differentiation medium for 3 weeks. SP cells cultured in a DMEM/F12 medium served as a control. As shown in Figure 1(g), both K7M2 and MG63 OSCs were found to have more calcium nodular and proteoglycan depositions than the non-SP cells, suggesting that the OSCs had undergone osteogenesis and chondrogenesis. These results indicated that the OSCs had the ability to differentiate into osteoblasts and chondrocytes. Furthermore, the pluripotent transcription factors Sox2 and Oct4 and CSC markers CD44, CD105, CD133, and Stro-1 were more highly expressed in both K7M2 and MG63 SP cells than in non-SP cells (Figure 1(h)), indicating that the SP cells have the characteristics of CSCs. Furthermore, we found that there is no difference in biomarkers in CD44, CD133, CD105, and Stro-1 between sphere-forming cells and SP cells from the K7M2 and MG63 ([Supplementary-material supplementary-material-1]). Therefore, in the following experiment, we used sphere-forming cells.

### 3.2. Metformin Induces Cell Cycle Arrest in K7M2 and MG63 OSCs

**Figure 2:**
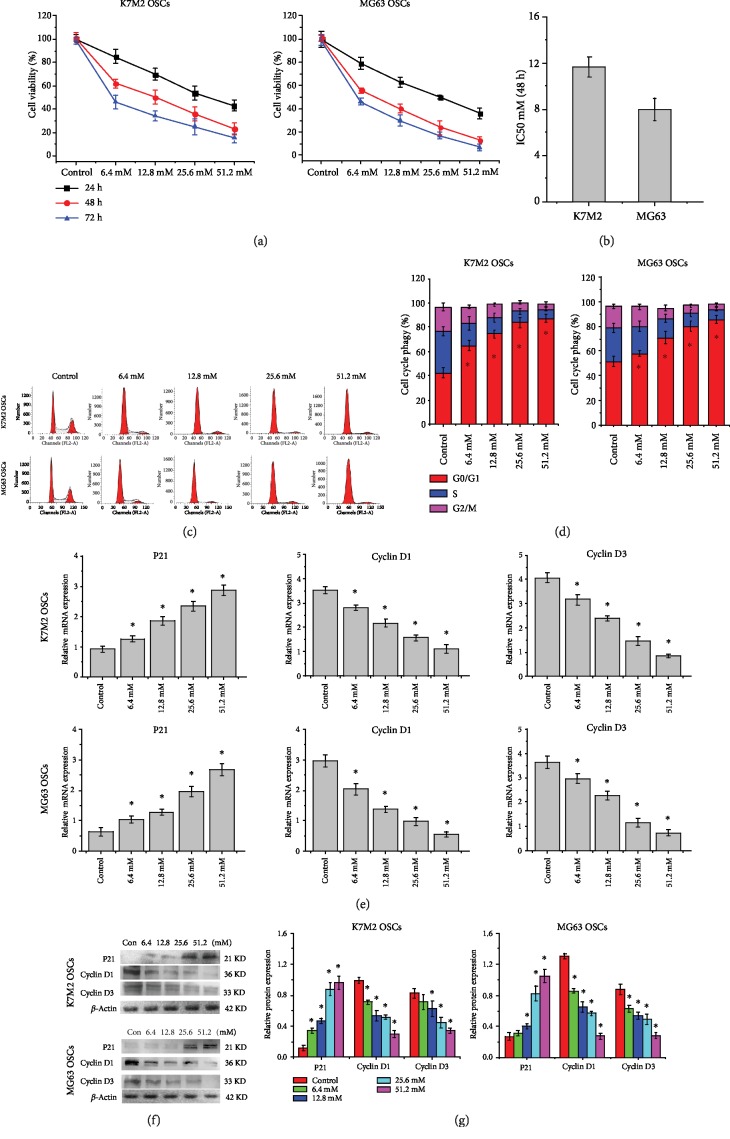
Metformin inhibits cell proliferation and induces G0/G1 arrest in OSCs. (a) The effect of metformin on the viability of K7M2 and MG63 OSCs by CCK-8. Cells were treated with 0, 6.4, 12.8, 25.6, or 51.2 mM of metformin for 24, 48, and 72 h. *n* = 3. (b) The IC50 of metformin in K7M2 and MG63 OSCs at 48 h. *n* = 3. (c) Cell cycle progression of K7M2 and MG63 OSCs treated with metformin. *n* = 3. (d) The percentage of cell cycle distribution in metformin-treated K7M2 and MG63 OSCs. (e) The mRNA expression levels of the cell cycle regulation genes *P21*, *Cyclin D1*, and *Cyclin D3* in K7M2 and MG63 OSCs. *n* = 3. (f) Western blot analysis of the cell cycle-related proteins p21, Cyclin D1, and Cyclin D3 in K7M2 and MG63 OSCs. *n* = 3. (g) Densitometric analyses of the cell cycle-related proteins. ^∗^*P* < 0.05 was considered statistically significant.

A dose- and time-dependent decrease in cell viability following metformin treatment was observed in Figure 2(a). The half-maximal inhibitory concentration (IC50) of metformin at 48 h was 11.8 ± 0.8 mM for the K7M2 OSCs and 7.9 ± 1.1 mM for the MG63 OSCs (Figure 2(b)). Flow cytometric analysis was used to examine the effect of metformin on the cell cycle. Treatment with increasing concentrations of metformin for 48 h resulted in the accumulation of cells in the G0/G1 phase and a decrease in the number of cells in the S phase (Figures 2(c) and 2(d)). Real-time PCR (Figure 2(e)) and western blot analysis (Figures 2(f) and 2(g)) clearly showed that the expression levels of cell cycle regulatory genes and proteins Cyclin D1 and Cyclin D3 were downregulated in both K7M2 and MG63 OSCs following metformin treatment, while P21 was upregulated. These results suggested that metformin induced cell cycle arrest in OSCs *in vitro* by blocking the G0 to G1 transition.

### 3.3. Metformin Activates a ROS-Mediated Mitochondrial Pathway to Induce Apoptosis

**Figure 3:**
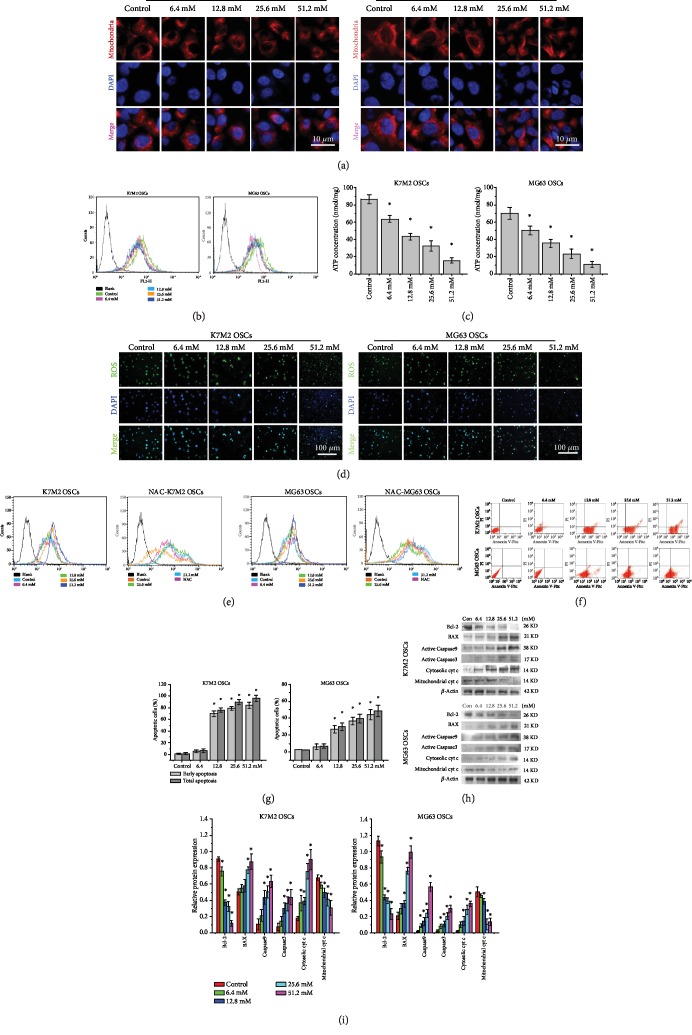
Metformin activates the mitochondrial pathway to induce apoptosis. (a) Confocal laser scanning microscope of the mitochondrial morphology in K7M2 and MG63 OSCs by Mito Tracker. Nuclei were counterstained with DAPI. Scale bars = 10 *μ*m. *n* = 3. (b) Flow cytometry analysis of mitochondrial membrane potential in K7M2 and MG63 OSCs exposed to metformin. *n* = 3. (c) ATP concentrations in K7M2 and MG63 OSCs after metformin treatment for 48 h. *n* = 3. (d) Immunofluorescence analysis of ROS in K7M2 and MG63 OSCs using the DCFH-DA probe. Nuclei were counterstained with DAPI. Scale bars = 100 *μ*m. *n* = 3. (e) Measurement of intracellular ROS in K7M2 and MG63 OSCs labeled with DCFH-DA by flow cytometry. Cells were pretreated with 10 mM of the ROS scavenger NAC to eliminate ROS. *n* = 3. (f) Annexin V-FITC/PI staining of apoptosis in metformin-mediated K7M2 and MG63 OSCs by flow cytometry. *n* = 3. (g) The percentage of OSCs in early apoptosis and total apoptosis upon treatment of metformin. (h) Western blot analysis of mitochondrial apoptotic pathway-related proteins in metformin-treated K7M2 and MG63 OSCs. *n* = 3. (i) Densitometric analyses of mitochondrial apoptotic pathway-related proteins. ^∗^*P* < 0.05 was considered statistically significant.

As apoptosis is often associated with mitochondrial function, we first assessed the effect of metformin on the mitochondrial morphology of the OSCs. Treatment with metformin for 48 h resulted in a change from the tubular network morphology to the disintegration of the mitochondrial network and reduced mitochondrial branching (Figure 3(a)). To evaluate the changes in the mitochondrial membrane potential as a result of metformin treatment, cells were subjected to Mito flow fluorescent dye. As shown in Figure 3(b), the metformin-treated cells decreased mitochondrial membrane polarization in a dose-dependent manner. Also, the intracellular ATP concentrations were declined dramatically following metformin treatment for 48 h (Figure 3(c)). We next explored whether the metformin-mediated apoptotic effect was a function of ROS modulation within the OSCs. As illustrated in Figure 3(d), immunofluorescence assay showed that OSCs treated with metformin for 48 h had higher fluorescent intensity compared to the controls, indicating an elevated ROS level upon metformin treatment. In addition, flow cytometric analysis in Figure 3(e) also indicated that metformin treatment for 48 h markedly increased ROS production in a dose-dependent manner. However, N-acetyl-L-cysteine (NAC) partly reversed the metformin-induced increase in ROS levels. Furthermore, the proapoptotic effects of metformin were evaluated by the FITC-Annexin V/PI apoptosis detection kit. As shown in Figures 3(f) and 3(g), metformin induced apoptosis in both K7M2 and MG63 OSCs in a dose-dependent manner. Western blot analysis (Figures 3(h) and 3(i)) showed that treatment with metformin decreased antiapoptotic Bcl-2 and increased proapoptotic Bax in both K7M2 and MG63 OSCs, confirming that metformin induced apoptosis in the OSCs. In addition, metformin was found to stimulate the translocation of cytochrome c from the mitochondria to the cytosol and upregulate both activated Caspase9 and activated Caspase3. Taken together, these results suggested that metformin induced apoptosis in OSCs mainly through a ROS-mediated mitochondrial dysfunction pathway.

### 3.4. Metformin Impairs Stemness and Pluripotency of OSCs

**Figure 4:**
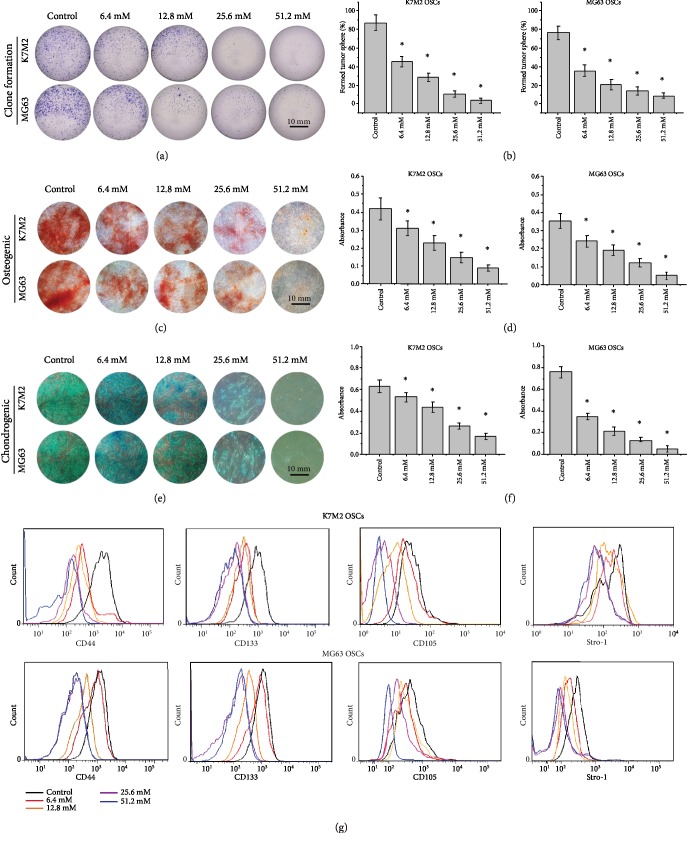
Metformin impairs stemness of OSCs. (a) Colony formation images of K7M2 and MG63 OSCs treated with metformin. Scale bars = 10 mm. *n* = 5. (b) Quantification of tumor spheres formed at day 7. (c) Osteogenic differentiation of K7M2 and MG63 OSCs treated with metformin. Scale bars = 10 mm. *n* = 3. (d) Semiquantification of the osteogenic differentiation capacity of K7M2 and MG63 OSCs by a spectrophotometer. (e) Chondrogenic differentiation of K7M2 and MG63 OSCs. Scale bars = 10 mm. *n* = 3. (f) Semiquantification of the chondrogenic differentiation capacity of K7M2 and MG63 OSCs by a spectrophotometer. (g) Flow cytometry of CSC surface markers CD44, CD105, CD133, and Stro-1 in K7M2 and MG63 OSCs. *n* = 3. ^∗^*P* < 0.05 was considered statistically significant.

Sphere formation assays were conducted to evaluate the stemness characteristics of the K7M2 and MG63 OSCs. The numbers of spheres significantly decreased in a dose-dependent manner following metformin treatment compared to the controls (Figures 4(a) and 4(b)). To investigate the effect of metformin on the pluripotency on the OSCs, the sphere-forming cells of K7M2 and MG63 were cultured in an osteogenic and chondrogenic induction differentiation medium with or without metformin. Osteogenesis (Figures 4(c) and 4(d)) and chondrogenesis (Figures 4(e) and 4(f)) were confirmed by the deposition of calcium and proteoglycans. The results showed that metformin reduced the pluripotency ability of the OSCs to differentiate into osteoblasts and chondrocytes in a dose-dependent manner. To further evaluate the effect of metformin on the stemness properties of the OSCs, the CSC markers CD44, CD105, CD133, and Stro-1 were also measured by flow cytometry (Figure 4(g)). As expected, the expression of CD44, CD105, CD133, and Stro-1 was downregulated in OSCs following metformin treatment in a dose-dependent manner, demonstrating that metformin may inhibit the stemness of OSCs.

### 3.5. Metformin Regulates Autophagy via the AMPK/mTOR Pathway

**Figure 5:**
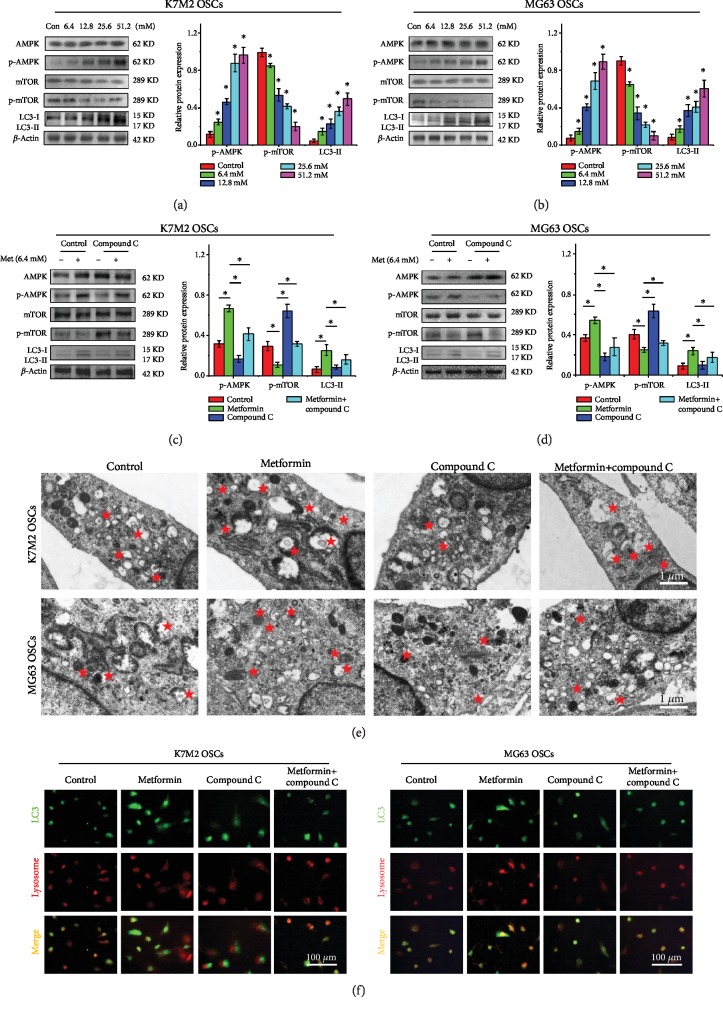
Metformin regulates autophagy via the AMPK/mTOR pathway. (a) Western blot analysis and (b) densitometric analyses of metformin on the AMPK pathway in K7M2 OSCs and MG63 OSCs. (c) Western blot and (d) densitometric analyses of compound C on metformin-mediated autophagy in K7M2 and MG63 OSCs. (e) TEM images of autophagosomes in the compound C-treated K7M2 and MG63 OSCs. The pentagrams stand for autophagosomes. Scale bars = 1 *μ*m. (f) Immunofluorescence assay of autophagy in the compound C-treated K7M2 and MG63 OSCs. Scale bars = 100 *μ*m. Each experiment was performed in triplicate. ^∗^*P* < 0.05 was considered statistically significant.

Metformin treatment resulted in a dose-dependent activation of AMPK phosphorylation (p-AMPK) in both the K7M2 OSCs (Figure 5(a)) and the MG63 OSCs (Figure 5(b)). Further investigation showed that metformin repressed the phosphorylation of mTOR (p-mTOR) and that this prolonged inactivation of mTOR and eventually induced autophagy. These results indicated that the AMPK signaling pathway might be involved in metformin-induced autophagy in K7M2 and MG63 OSCs. To further determine whether the AMPK signaling pathway is required for the induction of autophagy in response to metformin, K7M2 and MG63 OSCs were treated with an AMPK inhibitor, compound C. After treatment with 10 *μ*M compound C for 48 h, the metformin-induced autophagy of the K7M2 OSCs (Figure 5(c)) and MG63 OSCs (Figure 5(d)) was partially attenuated. In contrast to the effects of metformin, inhibition of AMPK activated the protein expression of p-mTOR and reduced the expression of LC3. TEM images (Figure 5(e)) and immunofluorescence assay (Figure 5(f)) further illustrated that inhibition of the AMPK signaling pathway by compound C attenuated the metformin-induced autophagy of both K7M2 OSCs and MG63 OSCs. Thus, our results indicated that metformin regulated the autophagy of OSCs via the AMPK/mTOR signaling pathway.

### 3.6. Autophagy Regulates Homeostasis of Pluripotency in OSCs

**Figure 6:**
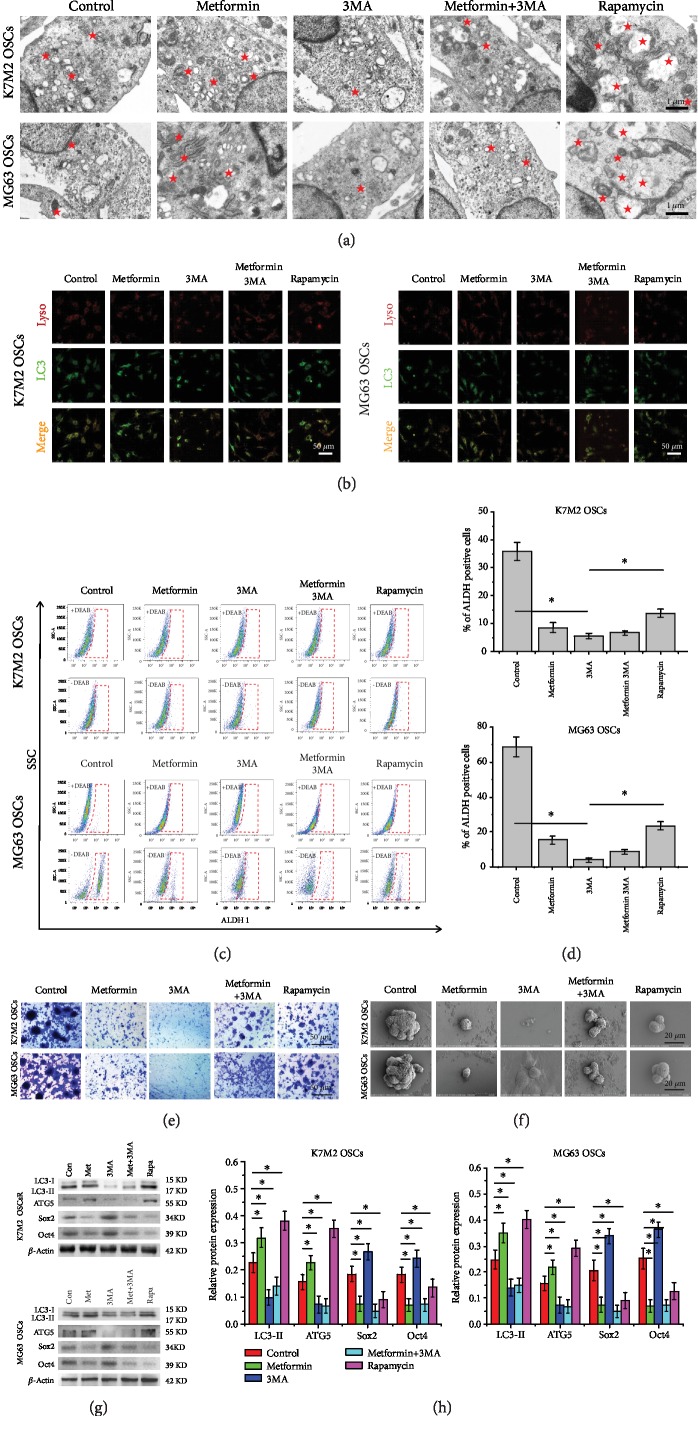
Autophagy regulates the homeostasis of pluripotency in OSCs. (a) TEM images of autophagosomes in metformin-treated K7M2 and MG63 OSCs. The pentagrams stand for autophagosomes. Scale bars = 1 *μ*m. (b) Immunofluorescence analysis of autophagy in K7M2 and MG63 OSCs. The colocalization (orange) staining of LC3 (green) with lysosome (red) indicates autophagy. Scale bars = 50 *μ*m. *n* = 3. (c) Flow cytometry analysis of the effect of autophagy on ALDH1 activity. The upper panel shows that OSCs were incubated with DEAB, a specific ALDH inhibitor. The lower panel shows ALDH1-positive OSCs. *n* = 3. (d) The percentage of ALDH1-positive OSCs. (e) Clone formation of K7M2 and MG63 OSCs. Scale bars = 50 *μ*m. *n* = 5. (f) SEM images of sphere forming of K7M2 and MG63 OSCs. Scale bars = 20 *μ*m. *n* = 3. (g) Western blot analysis of the autophagy markers ATG5 and ATG7 and the pluripotency-associated proteins Sox2 and Oct4 in K7M2 and MG63 OSCs. *n* = 3. (h) Densitometric analyses of autophagy markers and pluripotency-associated proteins. Each experiment was conducted at least three occasions. ^∗^*P* < 0.05 was considered statistically significant.

To verify whether the metformin-mediated autophagy is associated with the pluripotency and stemness of the OSCs, K7M2 and MG63 OSCs were treated with metformin, autophagy inhibitor 3MA, and metformin together with 3MA. The autophagy inhibitor 3MA alone served as a negative control, and the autophagy inducer rapamycin served as a positive control. Following treatment, markers of autophagy, stemness, and pluripotency were detected. Our results revealed that metformin induced autophagy, while metformin+3MA significantly alleviated the metformin-induced autophagy. Treatment with 3MA, the autophagy inhibitor, decreased the number of autophagosomes while autophagy inducer rapamycin promoted the occurrence of autophagy (Figure 6(a)). In addition, immunofluorescence expression levels of LC3 were elevated in OSCs treated with metformin or rapamycin, while treatment with 3MA inhibited the expression of LC3 as shown in Figure 6(b). In addition, the expression of ALDH1 of OSCs was decreased in the OSCs in all cases, no matter if the autophagy was enhanced (rapamycin or metformin) or impaired (3MA) as shown in Figures 6(c) and 6(d). The clone formation of OSCs was also assessed by crystal violet staining (Figure 6(e)) and SEM observations (Figure 6(f)). In agreement with the above results, both inhibition and induction of autophagy blocked the clone formation of the K7M2 and MG63 OSCs. These data indicated that autophagy might play a critical role in the maintenance of pluripotency of OSCs, since the pluripotency of stem cells was mainly maintained by the networks of pluripotency-associated transcription factors such as Oct4, Sox2, and Nanog. When autophagy was enhanced, the degradation of pluripotency-associated transcription factors was also enhanced, whereas autophagy inhibition caused an increase of pluripotency-associated transcription factors as shown in Figures 6(g) and 6(h). Although markers of pluripotency such as Sox2 and Oct4 were enhanced in OSCs when autophagy was inhibited, the tumor sphere formation and stemness were significantly attenuated (Figures 6(c)–6(f)). Given these results, we speculated that autophagy may regulate homeostasis of pluripotency in OSCs.

### 3.7. Metformin Inhibits Tumor Growth and OSC Capacities in a Mouse Xenograft Model

**Figure 7:**
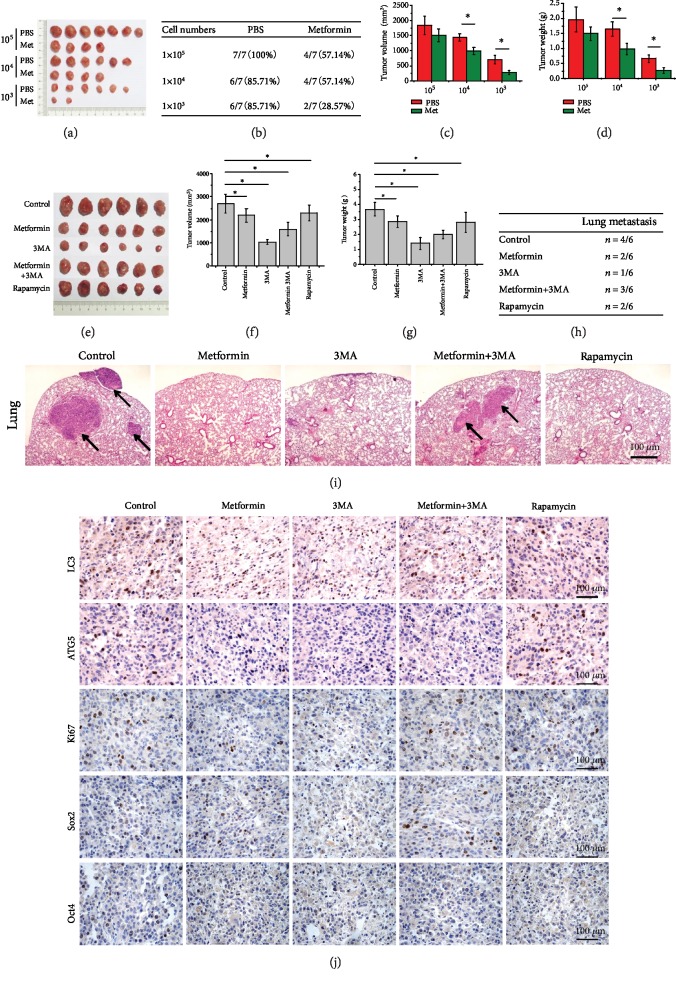
Metformin decreases K7M2 xenograft tumor growth and inhibits lung metastasis *in vivo*. (a) Metformin suppressed tumorigenicity of K7M2 OSCs *in vivo* by limited dilution assay. *n* = 7. (b) The tumor formation rate of K7M2 OSCs treated with metformin. (c) The tumor volumes and (d) tumor weights at the end of the experiments. (e) Images of resected tumor xenografts on day 21. *n* = 6. (f) The tumor volumes and (g) the tumor weights at the end of the experiments. (h) Numbers of mice with lung metastasis. (i) H&E staining of lung tissues for metastatic nodules. The arrows represent smaller tumor nodules in the lung. Scale bars = 100 *μ*m. (j) Immunohistochemical staining of LC3, ATG5, Ki67, Sox2, and Oct4 of three different tumors from each group. Scale bars = 100 *μ*m. ^∗^*P* < 0.05 was considered statistically significant.

Since 6.4 mM metformin could induce autophagy (Figure 5(a)) without causing apoptosis (Figure 3(f)) or death ([Supplementary-material supplementary-material-1]) of K7M2 OSCs, so we pretreated K7M2 OSCs with 6.4 mM metformin for 48 h to evaluate the tumorigenicity *in vivo*. As shown in Figures 7(a) and 7(b), K7M2 OSCs implanted at a cell density of 1 × 10^5^ formed tumors in 100% of control mice, whereas the tumor formation rate was reduced to 57.14% in metformin treatment. The tumor formation rate after implantation of 1 × 10^4^ OSCs were 85.71% in the control group and again 57.14% in the metformin-treated group. Implantation with 1000 cells resulted in a tumor formation rate of 85.71% in the control group; however, implantation at this density resulted in a sharp decrease in the tumor formation rate in the metformin-treated group to 28.57%. Moreover, metformin treatment significantly decreased the tumor volumes (Figure 7(c)) and weights (Figure 7(d)).

In order to determine whether metformin affected tumor growth *in vivo*, 1 × 10^5^ K7M2 OSCs were introduced into mice via intratibial injection. When the tumors reached approximately 100 mm^3^, the mice were randomly divided into the 5 groups described above (Figure 7(e)). After 3 weeks of treatment, the mice were executed, and the tumor tissues were stripped and weighed. Strikingly, the tumor volumes (Figure 7(f)) and tumor weights (Figure 7(g)) in all groups were significantly reduced compared with the control group. Lung metastases of the mice were also observed by visible metastatic nodules. There were significantly fewer mice with lung metastases in the metformin group than in the control group (Figure 7(h)). Metastatic nodules in the lungs were markedly decreased in the metformin-treated group (only 2 out of 6 mice) compared to the control group (4/6), indicating that metformin reduced the progression of osteosarcoma metastasis. This effect was also observed in mice treated with 3MA (1/6) and rapamycin (2/6). H&E staining of lung tissues (Figure 7(i)) further confirmed these findings that metformin- and rapamycin-induced autophagy suppressed the tumorigenicity of K7M2 OSCs *in vivo*. IHC staining (Figure 7(j)) revealed that both LC3 and ATG5 were upregulated, indicating that autophagy was induced in the tumors of mice treated with metformin compared to the control, whereas the expression of Ki67 was downregulated. Interestingly, Sox2 and Oct4 accumulated when autophagy was inhibited by 3MA and decreased when autophagy was induced by either metformin or rapamycin, which was consistent with the results *in vitro*.

## 4. Discussion

CSCs, also known as tumor-initiating cells, contribute to tumor initiation, progression, and metastasis [17]. The sphere formation assay is a classically widely used method to isolate and characterize CSCs [18]. Accumulating evidence has demonstrated that SP cells, which exhibit the characteristics of CSCs and are responsible for tumor metastasis and chemoradiotherapy resistance, are able to effectively exclude Hoechst 33342 dye [19]. Some pluripotent transcription factors such as Oct4, Sox2, and Nanog are essential to maintain the pluripotency and self-renewal of CSCs [20]. Furthermore, similar to pluripotent transcription factors, CD44 and CD133 are other established and ubiquitous CSC surface markers [21]. ALDH1, an enzyme located in the cytoplasm and mitochondria, has been identified as a predictive marker of OSC in cancer [22]. In this study, we used both tumor sphere culture and SP analysis to evaluate the characteristic of CSCs in K7M2 and MG63 osteosarcoma cell lines. We observed that the proportion of SP cells in K7M2 and MG63 was about 1%-2%, and this was reduced to about 0% after suppression by treatment with verapamil. Furthermore, both K7M2 and MG63 could form tumor spheres when cultured in a serum-free medium. Interestingly, we found that tumor spheres derived from K7M2 and MG63 osteosarcoma cells have higher indications of stemness, and the basal autophagy flux level was higher in tumor spheres than in their parental adherent cells, which is consistent with other studies that showed that autophagy regulated the stemness of CSCs [23]. When cultured in osteogenic and chondrogenic differentiation induction media, the OSCs differentiated into osteoblast-like and chondroblast-like cells. In correlation with the above research, the isolated OSCs were also positive for Sox2, Oct4, CD133, CD105, Stro-1, and ALDH1, whereas their parental cells were not.

Metformin, the first-line pharmacotherapy for type 2 diabetes [24], has recently emerged as a potential anticancer drug [25–27]. Since anticancer effect is usually concurrent with cell growth inhibition and cell cycle arrest, cell cycle deregulation is considered to be one of the hallmarks of tumor cells [28]. The vast majority of tumor cells have been found to have alterations in the cell cycle transition from G1 to S phase, which is mediated by cyclin-dependent kinase (CDK). p21, an inhibitor of CDK2, plays a critical role in G1/S transition [29]. This study demonstrated that metformin treatment activated the expression of *P21* gene and suppressed the expression of CDK genes such as *Cyclin D1* and *Cyclin D3*, resulting in the accumulation of OSCs halted in the G0/G1 phase. This observation is in agreement with the findings of Wang et al. [30], who showed that metformin blocked cell cycle progression in myeloma cells. More recently, Bao et al. [31] found that metformin weakens the migratory and invasive capacities of osteosarcoma cells *in vitro* and *in vivo*. In correlation with these results, mice treated with metformin had a significant decrease in tumor volumes compared to control mice in our study, confirming the antitumor effect of metformin.

Apoptosis, a programmed cell death apart from necrosis, plays a pivotal role in cancer therapy [32]. It is well known that apoptosis is usually driven by mitochondrial dysfunction, which results in the loss of the mitochondrial transmembrane potential and the accumulation of intercellular endogenous ROS [33], and excess ROS can oxidize DNA, proteins, and lipids and subsequently activate caspase-mediated apoptosis [34]. We confirmed that metformin treatment triggered excessive ROS production, while pretreatment with NAC partly reversed the metformin-induced accumulation of ROS in OSCs. Particularly, the loss of the mitochondrial transmembrane potential induced an imbalance between Bcl-2 and Bax. Bax, a proapoptotic protein, translocated to mitochondria and thereby resulted in the release of cytochrome c [35]. In correlation with these results, metformin treatment induced mitochondrial dysfunction accompanied by the loss of mitochondrial transmembrane potential, as well as decreased the expression of antiapoptotic protein Bcl-2 and increased the activities of Caspase9 and Caspase3.

There is accumulating evidence demonstrating that AMPK signaling could suppress the proliferation of cancer cells and enhance their CSC properties [36]. In addition, metformin has been known to suppress the self-renewal ability of CSCs in colorectal cancer, dependent on the relative regulation of glutamine metabolism [37]. Consistent with these findings, we showed that metformin substantially reduced the pluripotency of K7M2 and MG63 OSCs through the inhibition of ATP synthesis in mitochondria and the activation of AMPK. This is also in line with the finding that autophagy is regulated by the AMPK/mTOR pathway [38]. Moreover, the AMPK inhibitor, compound C, and the autophagy inhibitor, 3MA, were applied to study the roles of the AMPK/mTOR pathway in autophagy [39]. Our results showed that activation of AMPK inhibited the mTOR pathway, in which the autophagy seemed to be enhanced.

Autophagy, a lysosomal degradation pathway involved in the maintenance of cellular homeostasis is emerging as an attractive therapeutic target in certain types of tumors [40]. Several methods exist to induce autophagy in cells, including starvation and treatment with rapamycin [41]. In this study, we found that the autophagy markers LC3, ATG5, and ATG7 were notably increased in OSCs treated with metformin, indicating that metformin can stimulate autophagy, which is consistent with the results of Wang et al.'s study [30] in myeloma cells. Using this model, we first examined the viability of OSCs and found that it decreased upon treatment with metformin. Moreover, the expression of stem cell markers Oct4 and Sox2 was decreased when autophagy was induced by metformin or rapamycin, indicating that autophagy might help to maintain the pluripotency of OSC. Finally, we confirmed the direct influence of autophagy on the capacity of the OSCs to self-renew by suppressing autophagy with 3MA. Although the potential stem cell markers Oct4 and Sox2 were more highly expressed in cells treated with 3MA, however, the cloning capacity of OSCs was weakened. Furthermore, the metformin-mediated reduction in the number of spheres observed could be partially reversed by 3MA treatment, suggesting that autophagy is essential for the maintenance of pluripotency in OSCs. Similarly, it has been demonstrated that autophagy regulates the homeostasis of pluripotency-associated proteins in human embryonic stem cells [42], and imbalances in these proteins may lead to the loss of pluripotency in embryonic stem cells [43]. In the present study, we demonstrated for the first time that metformin suppresses self-renewal ability and tumorigenicity of OSCs by targeting autophagy, and autophagy regulates homeostasis of pluripotency of OSCs.

## 5. Conclusions

**Figure 8:**
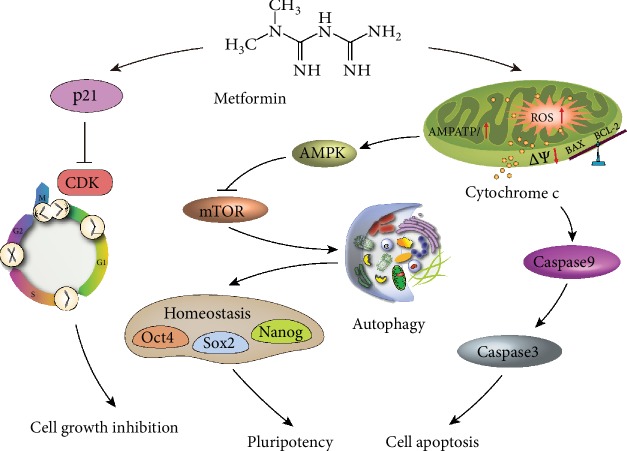
Proposed mechanisms of metformin on osteosarcoma. Metformin significantly induced G0/G1 phase arrest by blocking the activity of cyclin-dependent kinases. Metformin also resulted in apoptosis via a ROS-dependent mitochondrial pathway. Moreover, metformin induced autophagy via the AMPK/mTOR pathway and then disrupted the homeostasis of stemness and pluripotency of OSCs.

In conclusion, we found that treatment with metformin inhibited the proliferation of K7M2 and MG63 OSCs by inducing G0/G1 phase cell cycle arrest. In addition, metformin simultaneously triggered apoptosis in OSCs, which promoted cell death via a ROS-dependent mitochondria-mediated pathway. Strikingly, metformin significantly suppressed the self-renewal ability and tumorigenicity of OSCs by regulating autophagy, which was modulated by the AMPK/mTOR pathway (Figure 8). Taken together, these data indicated that metformin exerted antitumor effects against OSCs and that autophagy may be a promising therapeutic target for osteosarcoma.

## Data Availability

The data used to support the findings of this study are available from the corresponding author upon request.

## Supplementary Materials

Supplementary 1 Figure S1: flow-cytometric analysis of biomarkers in CD44, CD133, CD105, and Stro-1 between sphere-forming cells and SP cells. Both the K7M2 and MG63 OSCs were positive for CSC markers CD44, CD133, CD105, and Stro-1. Proper isotype antibodies were used as a control (black lines).

Supplementary 2 Figure S2: AO/PI staining of K7M2 OSCs. Living cells are stained with AO (green), while dead cells are with PI (red). There is no significant difference in death after metformin incubation. AO: acridine orange; PI: propidium iodide. Scale bar = 100 *μ*m.
