# Supplementary figures and images for: Metformin Suppresses Self-Renewal Ability and Tumorigenicity of Osteosarcoma Stem Cells via Reactive Oxygen Species-Mediated Apoptosis and Autophagy
Source: Oxid Med Cell Longev. 2019 Nov 18;2019:9290728. doi: 10.1155/2019/9290728 (PMC6885828; doi:10.1155/2019/9290728)

## Slide 1
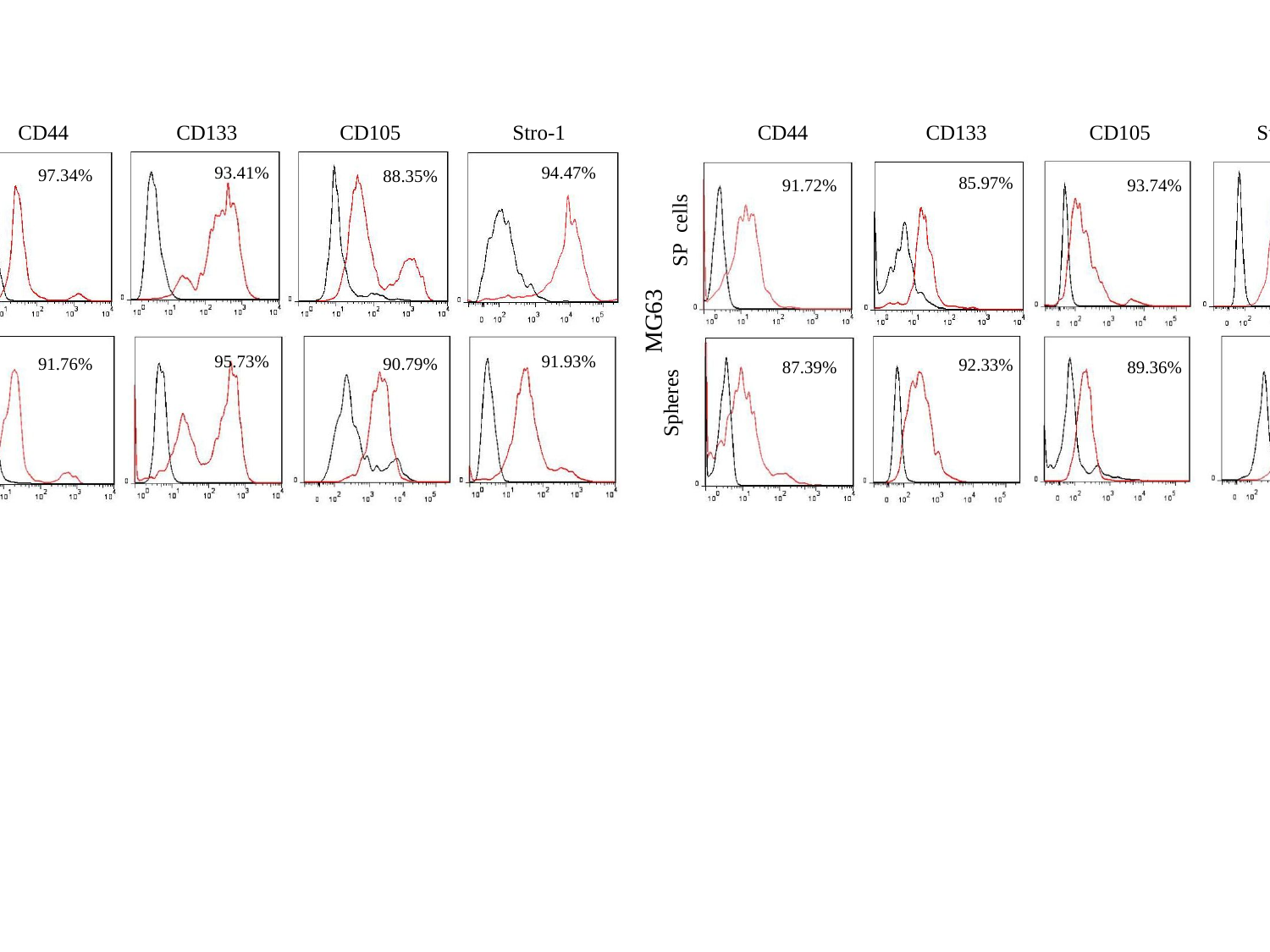

CD44
CD133
CD105
Stro-1
CD44
CD133
CD105
Stro-1
93.41%
94.47%
97.34%
88.35%
85.97%
97.86%
91.72%
93.74%
 SP cells
 SP cells
MG63
K7M2
95.73%
91.93%
91.76%
90.79%
92.33%
93.71%
87.39%
89.36%
Spheres
Spheres

Supplement: Supplementary 1 — Figure S1: flow-cytometric analysis of biomarkers in CD44, CD133, CD105, and Stro-1 between sphere-forming cells and SP cells. Both the K7M2 and MG63 OSCs were positive for CSC markers CD44, CD133, CD105, and Stro-1. Proper isotype antibodies were used as a control (black lines). [file 9290728.f1.pptx]

## Slide 1
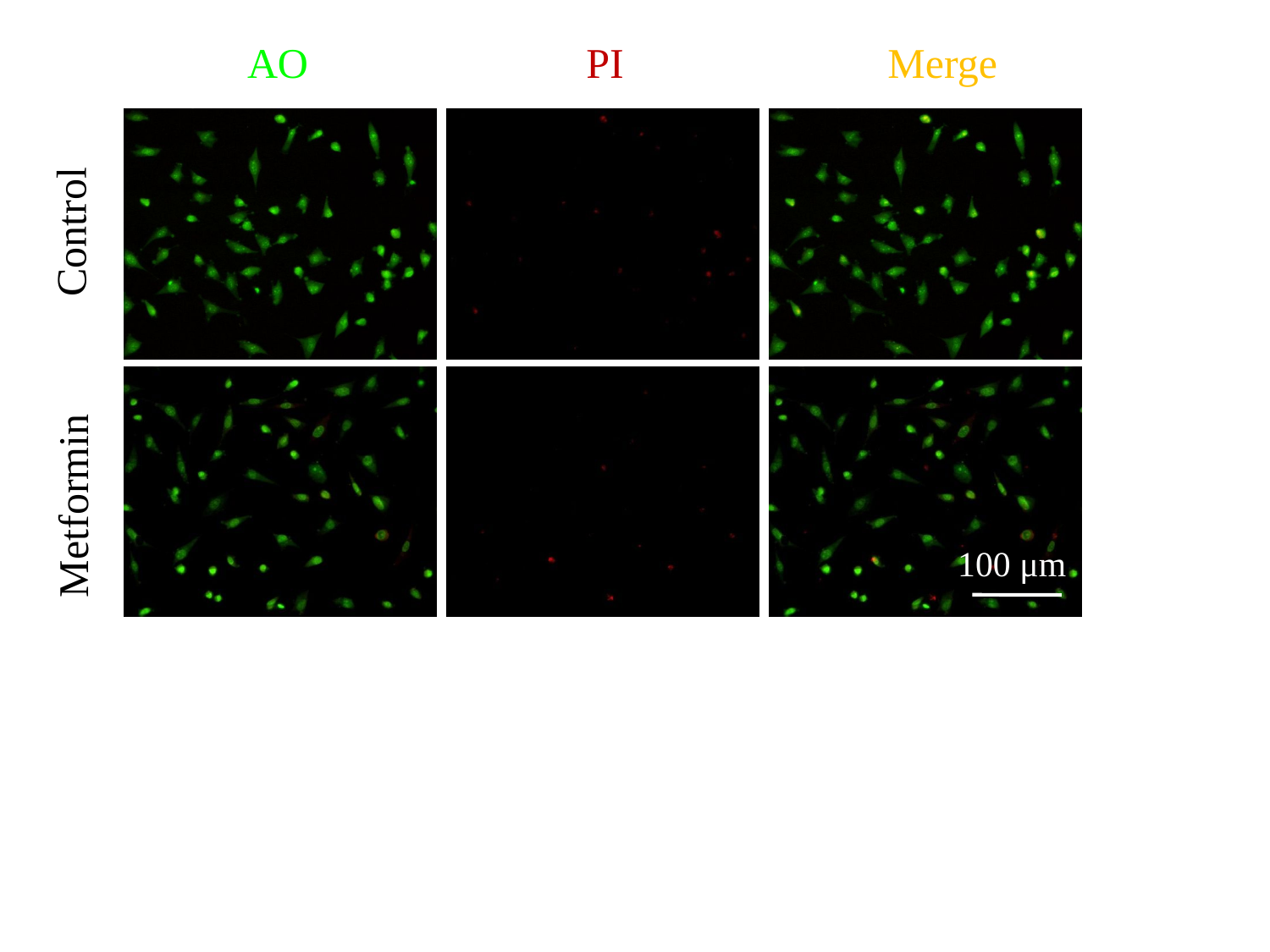

AO
PI
Merge
Control
Metformin
100 μm

Supplement: Supplementary 2 — Figure S2: AO/PI staining of K7M2 OSCs. Living cells are stained with AO (green), while dead cells are with PI (red). There is no significant difference in death after metformin incubation. AO: acridine orange; PI: propidium iodide. Scale bar = 100 μm. [file 9290728.f2.pptx]
